# Commercial Surrogacy Is not a Secret Handshake: It Is a High‐Five: Gay Fathers in China’s Changing Landscape

**DOI:** 10.1111/1468-4446.13214

**Published:** 2025-04-12

**Authors:** Eileen Y. H. Tsang

**Affiliations:** ^1^ Department of Social and Behavioural Sciences City University of Hong Kong Kowloon Hong Kong

**Keywords:** China, commercial surrogacy, fatherhood, field theory, upper‐class gay father

## Abstract

In 2023, China introduced regulatory amendments to birth certificates and the *hukou* (household registration) system, aiming to boost birth rates and offset an aging population. However, the implications of these changes on marriage and family support amidst population policy shifts remain underexplored. One particular area is how commercial surrogacy (CS) impacts gay communities where couples seek surrogate children to maintain intergenerational bonding and bridging within their familial and kinship networks. This article employs Bourdieu's field theory, characterized by class‐based capital and habitus, to examine how upper‐class gay individuals navigate this changing field in the three municipal cities of Beijing, Shanghai, and Tianjin. Drawing on in‐depth interviews with 35 upper‐class gay fathers and 21 gays' parents, their agency and changes in pursuing parenthood through transnational and underground CS in China are illustrated. This article posits that the formation of the family is inherently tied to the class‐based cultural capital and habitus of the gay individuals. The reproductive decision‐making process within the gay community reflect strategies to form families are imbued with class‐based capital and habitus.

## 1 Introduction

The practice of commercial surrogacy (hereafter CS) involves hiring a woman to carry a pregnancy for another individual or couple, essentially offering her womb as a service. The surrogate mother facilitates childbirth, ‘renting’ her womb as a profit‐making experience. CS is considered different from altruistic surrogacy, which tends to be motivated by benevolent, not‐for‐profit reasons (Rudrappa and Collins 2015; Pande 2014; Jacobson 2016; Smietana 2017). Despite being illegal in China, CS has seen a surge in reported cases, ranging from 60,000 to 100,000 between 2018 and 2023 (Qi et al. 2023). Affluent gay communities opt for CS rather than other forms of artificial reproductive technology. A burgeoning corpus of scholarly work chronicles the experiences of queer parents in Western nations (Dempsey 2016; Fantus 2021; Goodfellow 2015; Green et al. 2019; Rubio et al. 2020). This literature encompasses a broad range of topics, including parenting methodologies, child welfare, relationships with surrogates and egg donors, coming out experience, and comparative studies between homosexual father families, heterosexual families, and single father families (Fantus 2021; Goodfellow 2015).

In China, LGBTQ + studies explore the dynamics of “cooperative marriages” (*xinghun*) between gay men and lesbian women, as well as “marriages of convenience” between closeted gay men and *tongzhi* (heterosexual wives) (Tsang 2021a; Cheung and Tsang 2023). With the arrival of children, tensions arise as each individual navigates traditional Confucian family values (Bao 2020; Engebretsen 2014; Kam 2020). Recent research adopts dialectical family imaginaries (Lo 2023), and the neo‐Confucian homonormativity for queer communities to achieve harmonious family relationships (Luo et al. 2022) but both ignoring the policy changes. In January 2023, the government drastically altered restrictions on birth certificates to address several ongoing social issues. Birth certificates may now be issued to children irrespective of their parent's marital status or sexual orientation, including heterosexual, LGBTQ+, or even single parents. These changes represent a significant milestone in alleviating societal pressures on young adult Chinese men and women to procreate.

In this article, I employ Bourdieu's field theory to analyse the motivations behind upper‐class gay individuals with professional occupations and higher educational backgrounds who desire to have children. Bourdieu's field theory allows exploration of the interplay between individual agency and structural constraints to help us better understand the ways different forms of capital are valued, accumulated, and used within specific social fields. Applying field theory highlights the negotiation strategies of gay fathers as they draw upon their class‐based capital and habitus to benefit from the government's relaxed birth certificate regulations (Choi 2022; Loa and Choi 2024). This enables them to pursue CS services both domestically and internationally without the prerequisite of marriage. Examining field theory through the perspectives of gay and their parents can reveal how these individuals navigate fatherhood within a patriarchal society that stigmatizes gay communities. Three research questions undergird this study: (1) Why do some upper‐class gay individuals opt for CS? (2) What motivates the parents to support their gay sons in preparing for parenthood? (3) How do both upper‐class gay men and their parents utilize their class‐based capital and habitus in the education of their surrogate children or grandchildren? (4) How has the evolving landscape of birth certificate regulations facilitated gay parenthood in China since 2023?

These questions are addressed through field theory to interpret ethnographic data collected over a year and a half through interviews with 35 gay individuals and 21 parents in Beijing, Shanghai, and Tianjin. This article aims to identify the implications and connections between gay individuals having children through CS, and to understand how class‐based capital helps them educate and nurture their children within upper‐class families, with the support of the gay individuals' parents.

## 2 Bourdieusian Cultural Turn‐Field Theory

In Western countries, the dominant literature on gay fathers and parenting focuses on the well‐being of their children, relationships with surrogates and egg donors, and comparisons between gay‐father families, heterosexual families, and single‐father families in psychology and social work (Dempsey 2016; Fantus 2021; Green et al. 2019; Zeitlyn 2012). The decision to become parents often relates to their family relationships and their coming‐out experiences (Goodfellow 2015). Queer kinship and families are formed in various ways, including co‐parenting, adoption, and alternative kin ties (Tsang 2021a). The growing literature on gay fatherhood practices and CS pursuits among gay men has emphasized the impact of these decisions on their relationships and life choices (Golombok 2020; Smietana et al. 2014). Gay couples often pursue reproductive rights through different types of artificial reproductive technologies (ART) rather than traditional male‐female procreation activity (Mizielinska 2022).

ART have significantly transformed family formation, challenging traditional notions of kinship and familial structures across various cultural contexts. In Western societies, ART has redefined parenthood, disrupting conventional roles of motherhood and fatherhood (Thompson 2005). The procedures enable any person (or two people) to become a parent (or parents). Thus, Franklin and McKinnon (2001) contend that ART necessitates a rethinking of kinship, moving beyond biological connections to embrace more inclusive family models. This shift has spawned contentious debates over access, regulation, and myriad ethical implications, particularly concerning equity and justice (Ross and Moll 2020). Schadler (2016) emphasizes that a reconceptualization of family is needed to account for the dynamic interplay of material and technological influences since ART fosters ever‐evolving familial forms that defy static definitions. The globalization of ART, as discussed by Inhorn and Birenbaum (2008), underscores its transnational impact, influencing family structures and kinship norms across diverse cultural landscapes. Mamo (2007) notes that ART within LGBTQ + communities challenges heteronormative family paradigms and fosters new possibilities for family formation. Bradway and Freeman (2022) also highlight how race, sexuality, and kinship intersect within queer communities to challenge hegemonic family paradigms. While these studies predominantly reflect a Western perspective, Chinese queer communities face uniquely East Asian challenges regarding parenthood. How their parents support and help them adds a layer of complexity to the phenomenon (Chen 2024; Lo 2023; Yu et al. 2018).

In non‐Western countries, the existing research highlights the practice of “*xinghun”* or marriage of convenience between gays and *tongqi* (Tsang 2021a), resulting in marriage fraud, but which is a coping strategy for sexual minorities in East Asian societies (Brainer 2019). Societal expectations necessitate that they rarely disclose their sexual orientation, because coming out often creates family disharmony and tension. The discourse revolves around the transformation, subversion, concealment, and persistence of patriarchy and Confucian family values (X. Qi 2016). The CS literature indicates that affluent Chinese parents often opt for surrogate children through legal services in the US (Sahakian 2023), sometimes leading to legal disputes between intended parents and surrogate mothers (Y. Liu et al. 2022). Notably, Yang (2015) detailed the dark side of “informal surrogacy” (using friends, family members, or co‐workers as surrogate mothers), which can exploit the surrogates. Lo (2023) mentioned dialectical family imaginaries where lesbians navigate themselves, their community, and policy to achieve parenthood. Han explores how Chinese queer parents' participation in assisted reproduction has destabilized the dominant hetero‐reproductive family matrix. Chen (2024) identified the positive experiences of gay couples becoming parents through surrogacy in Taiwan. Recently, Luo et al. (2022) coined the phrase “neo‐Confucian homonormativity” to characterize a harmonious relationship between gay men and their families of origin to enter a monogamous relationship and become parents. T. T. Liu et al. (2024) reveal the intricate interweaving of cultural, political, and social factors that shape public anti‐surrogacy sentiment, particularly through the lens of social media, highlighting the cultural specificity of ART debates. Following values of neo‐familism, the support from their parents who used CS to have children are prominent in today's China (Wei and Yan 2021, 455). However, their research disproportionally focused on the difficulties of surviving under heterosexual norms and the relaxation of children's certification requirements since 2023.

The existing literature largely ignores the institutional trajectories and policy changes that intertwine with China's policy changes and the relational and dialectic relationships with their families of origin. Bourdieu's field theory provides a framework for understanding gay parenthood in Beijing, Tianjin, and Shanghai. It explores the interplay between individual agency and structural constraints and the ways different forms of capital are valued, accumulated, and used within specific social fields to react to the recent policy change on birth certificates.

Cultural capital comes in various forms, such as embodied, objectified, and institutionalized forms (Bourdieu 1977, 1989). Cultural capital comprises personal skills and social competencies such as flexibility, teamwork, and adaptability, which enhance employability in cultures that treasure skills like communication, persuasiveness, drive, resilience, adaptability, self‐confidence, and problem‐solving. Education, in this context, enables the class to progressively seize the means of cultural reproduction as a strategy to enhance its likelihood of social reproduction (Bourdieu 1977, 1989). In short, education is crucial to maintaining high social status from generation to generation. Habitus guides individuals in social life, and upper‐class gay individuals possess a distinctive habitus and taste in everyday life because of higher‐than‐average cultural capital. This process focuses on the substantive ingredients of cultural capital developed from education rather than the image conveyed by educational credentials (Igarashi and Saito 2014; Tsang 2020a). These self‐identified gay individuals often gravitate towards prestigious educational institutions for the acquisition of cultural capital, then move to cosmopolitan cities in China that offer lucrative career and entrepreneurial opportunities. This legitimizes their superior position relative to other classes.

Habitus can be defined as a system of durably acquired schemes of perception, thought, and action, engendered by objective conditions but tending to persist even after an alteration of those conditions' (Bourdieu and Passeron 1979, 12). In other words, habitus is a set of subjective dispositions that reflect a class‐based social grammar of taste, knowledge, and behaviour permanently inscribed in each developing person (Bourdieu 1977, 1989). Habitus is the key to class reproduction, forming a vital part of gay individuals' everyday life, thereby giving rise to hopes for landing a job and creating their class‐based sophistication and temperament based on their cultural capital (Tsang 2021b).

A field, as conceptualized by Bourdieu (1984), is understanding the “rules of the game,” which literally means controlling the games by knowing the guidelines for playing and winning each game. These fields are characterized by social differentiation and analytical autonomy, implying that each field is distinct and independent in its ideologies, beliefs, and rules (Bourdieu and Wacquant 1992; Bourdieu 1986). Drawing parallels with a game, Bourdieu (1982/1991) posits that every field has its unique objectives and a specific set of principles that are considered legitimate and coherent within that field. These principles are binding on all agents and entities that enter the field. The actors within a field implicitly agree on these rules and the stakes involved (Bourdieu and Wacquant 1992). To navigate and succeed within a field, actors utilize these rules to comprehend the social world and guide their actions in accordance with the structure of the field. The more cultural capital and inherent habitus, the more likely the individual will understand the “rules of the game” (Bourdieu 1991).

The cultural capital of the upper‐class gay individuals, characterized by their higher education, professional status, and able to obtain their overseas passports to relocate overseas, is significant. CS services are expensive and can require international travel. The neoliberal economy in China creates a domesticated and consumerist gay identity (Valocchi 2017). The habitus of gay individuals is shaped by their capital. The lifestyle, preferences, and dispositions that form their habitus are influenced by their social, cultural, and financial capital. For example, their habitus reflects how they can form a functional gay family without marriage and raise well‐adjusted children in a society that traditionally shuns non‐heteronormative family structures. The class‐based capital produces a specific habitus through their lifestyle and experiences, which in turn influences their family and their parents (Tsang 2014). Field provides an appropriate framework to discuss the experiences of these gay fathers and their surrogate children. The field, in this context, refers to the policy changes that relaxed the rules around birth certificates for surrogate children. This field is characterized by its unique set of rules, norms, and beliefs. Gay fathers, well‐versed in the rules of the game, may now choose to have surrogate children.

Spurred by Bourdieu's field theory, this article argues that gay individuals becoming fathers is inextricably linked to their class‐based cultural capital and habitus. Also, the reproductive decisions of aspiring gay parents are intricately entwined within a broader web of dialectical, relational, connected, and embedded relationships, particularly with their parents and family of origin.

## 3 Background: From One‐Child‐Policy to Lifting the Birth Rate

A confluence of factors has catalysed the demand for CS in China, including escalating infertility rates, relaxation of the one‐child‐per‐family policy, and a deeply ingrained cultural imperative to procreate. Greenhalgh (2008) examines the profound social and cultural impacts, including altered family dynamics and gender relations, critiquing the coercive implementation and human rights issues associated with these policies. Her work also addresses recent policy shifts, such as the two‐child policy, and their potential demographic implications. China's natalist policies have evolved significantly since 1949, reflecting shifting state priorities. Initially, the government encouraged population growth to strengthen the nation, but by the late 1970s, concerns over resource scarcity and economic development prompted a shift to anti‐natalist measures, culminating in the one‐child policy (Greenhalgh and Winckler 2005). This policy, implemented in 1979, aimed to control population growth through strict family planning regulations. Over time, the policy faced criticism for its coercive methods and social consequences, such as gender imbalances and producing a proportionally older population. In response, China introduced the two‐child policy in 2015, marking a significant relaxation of its population control measures to address those challenges.

In January 2023, the Chinese government further addressed demographic imbalances and declining birth rates by revising birth certificate policies. For example, the Guangdong Provincial Health Commission expanded birth registration eligibility beyond local household registration (*hukou*), allowing those residing anywhere in the province to register their children (Si 2023). Other provinces have followed suit, relaxing restrictions on the number of children eligible for a family to register. The registration can be either at the *hukou* location or the current residence. These policy changes have legitimized CS by allowing CS children to have a legal *hukou,* fuelling the rapid growth of black‐market CS services in China (Global Market Insights 2022). This black market will reportedly yield over 50,000 births annually (Global Market Insights 2022), both within China and internationally. A low‐end agency can covertly handle over 300 surrogacy cases annually, charging from US$10,000‐US$20,000 per child. A high‐end, black‐market agency in Shanghai offered packages starting from US$21,000 to US$50,000 per child and handled around 200 illegal cases annually. These vast sums of money attract physicians, surrogates, and other stakeholders to the CS black market.

Although CS remains illegal in China, up to 500 CS agencies operate nationwide (Everingham and Whittaker 2023), resulting in an estimated 60,000–100,000 cases of illegal CS per year (Qi et al. 2023). The CS market in China was estimated at over US$14 billion in 2022, up from US$6 billion in 2018 (Global Market Insights 2022; Qi et al. 2023). Meanwhile, China's overall population continues to decline, with a negative growth rate of −0.6% (National Bureau of Statistics of China 2022). Infertility is a prevalent issue in China, with one in every eight couples over age 35 experiencing difficulties conceiving (Gao et al. 2016). The number of infertility patients in the country exceeds 50 million, a staggering statistic in 2022 (Qi et al. 2023). Between 2007 and 2020, the infertility rate rose from 12% to 18%, indicating that approximately one in every 5.6 couples of childbearing age in China encounters conception difficulties (Li 2022).

## 4 Research Method: Interviewing Upper‐Class Gay Individuals

To explore this phenomenon, 35 self‐identified gay men in Shanghai, Beijing, and Tianjin were interviewed between January 2, 2023, and March 31, 2024. The recruitment strategy leveraged personal networks and recommendations from a Tianjin‐based gay NGO and another LGBTQ + NGO, Parents, Families, and Friends of Lesbians and Gays (PFFLG) in Shanghai. Additional participants were recruited via Shanghai Pride, Blued, Aloha, Jack'd, WeChat, Douyin, and other social media platforms. Most of the participants were highly educated, with 26 having bachelor's degrees, 5 earning master's degrees, and 4 holding doctorates in STEM fields. Most could be considered upper class, with a median annual income of US$154,000, nearly ten times the average median income in those cities. Another criterion for selecting the samples was that they must have obtained at least a bachelor's degree from a prestigious domestic or international university. Additionally, they must have at least one child when they accepted the interviews. Regarding occupational categories, 21 were entrepreneurs, 9 worked in professional fields, and 5 were cadres. All participants had disclosed their sexual orientation to their parents during their teenage years, or their parents were already aware that their sons were gay and had accepted them. Each participant had at least one child, with five participants having two children. The children ranged in age from 2 months to 10 years old. Among the five participants with two children, their first child was born before the relaxation of the birth certificate. Five participants were in long‐term relationships with their partners and were jointly raising their children. The remaining participants were single. None had entered into a marriage with a lesbian or a heterosexual woman.

This study also interviewed 16 mothers and 5 fathers of gays, aged 55–67, all holding at least a bachelor's degree. All of the mothers were retired. Before retirement, 11 were professionals, 2 were cadres, and 3 were entrepreneurs. To ensure participant privacy and research validity, informed consent was obtained, with participants briefed on the study's purpose and given the right to withdraw.

Interviews were semi‐structured, focusing on parenthood motivations, CS processes, and child‐rearing strategies within a gay family context. Informal engagement methods, such as WeChat and Tencent Meeting, were used. Interactions with informants occurred at their homes and family gatherings. This approach enriched the data, providing insights into the informants' daily lives and personal histories (Bernard 2011). Data, including recorded interviews, in situ notes, and post‐event field notes, were transcribed and then translated from Putonghua to English and uploaded into NVivo 12.0. Given the scope of this paper, the verbatim excerpts selected are based on the conceptual framework of field theory and serve as representative illustrations of the research questions.

The initial analysis was guided by the field theory, which generated additional themes and the research questions. These were categorized and re‐coded using the grounded theory method (Strauss and Corbin 1990), with axial coding employed to compare and categorize different codes and identify subordinate themes. Selective coding comparisons generated superordinate themes, informing the theoretical implications of the findings. Pseudonyms and slightly modified biographies were used to protect participant identities. The translations sought to maintain the spirit and intention of the informants' viewpoints. The study was approved by the investigator's host university Institutional Review Board.

Given the relatively small sample size, the findings cannot be generalized to gay populations across China. Another limitation is that many of the participants were upper class with professionals, cadres, and entrepreneurs with cultural capital, social networks, and studying overseas experience. This article thus represents a significant step forward in opening a more comprehensive discussion of diverse views and experiences regarding family formation and having children in gay communities following the announcement of birth certificate policy changes.

## 5 Upper‐Class Gay Individuals' Class‐Based Capital and Habitus

The possession of class‐based cultural capital by the upper‐class gays not only aids them in securing lucrative employment but also facilitates their navigation through various socio‐cultural cultivations and practices for their surrogate children. A recurring theme in the data is the emphasis on social status and cultural capital. Many participants highlighted the importance of providing their children with high‐quality education and opportunities. This pattern suggests that the desire to have children is intertwined with maintaining and enhancing social status. A quintessential example of high economic capital is their capacity to employ a nanny and garner support from their parents. Nilan was a 34‐year‐old who spent two decades studying in the United States and is currently employed at a Shanghai investment bank. His parents have been aware of his sexual orientation since he was 13 years old. They sent him to the USA and paid or him to study risk management. Nilan's parents respect his choices as he earns a good salary and has a prestigious job. After China relaxed the birth certificate policy, Nilan and his partner decided to pursue CS to have a child. Their daughter was born in LA and was 3 months old at the time of the interview. Nilan's education, career success, and financial stability serve as capital that enabled him to use legal CS in the USA. Their robust cultural capital provided the couple with a buffer, and their affluent family background also lent support. Nilan says,

This is not something we were forced into; it was a choice we made willingly. We have enough money to have a surrogate child in the USA! We appear to be a typical family, don’t we? The first time I laid eyes on my baby girl, I was struck by how much her eyes resembled mine. Since I am successful my parents usually let me do whatever I want. If I were poor, perhaps they might force me to trick a girl into getting married. But I have always adored children, and my childhood dream was for my future child to wake up in the middle of the night and see me smiling.

Jun (36), resides in Tianjin, and he is a cadre. He vividly mentioned that his educational background and class‐based capital augments his personal legacy and joy to have a baby through CS.

I would love to become a dad, and my stable and high‐income job can support me to have transnational surrogacy in the USA. I am solo for my baby boy. I do not have a partner to join my family. However, my heart nudges me toward fatherhood. It is not obedience; it is a joy. I know the steps—the lullabies, the diaper ballet. And I want to waltz, even if it is a solo act. Legacy is not a duet; it is a chorus of memories echoing through generations.

Most of the gay fathers have a strong sociocultural perspective on how to educate and socialize their children. They are committed to providing what society considers a ‘normal’ family for children. The baby has become a bridge, connecting the family, relatives, and kinship. Typically, most gay fathers take on the role of the father, while their partners, mothers, or nannies assume the role of the mother. However, they are aware of their limitations. Peter says,

It was beyond belief. Neither my partner nor I can really provide the traditional maternal care that our kid might need. But we are doing our best, and that is what counts, right? My partner, bless him, he has got this gentle, patient side that really shines. He is the one who tucks our little one in at night, tells bedtime stories, helps with homework, and even does the shopping. And his cooking? Absolutely delicious! As for me, well, I am more of the ‘get‐things‐done’ type. I am the one who makes sure our kid gets to school on time and teaches him the ins and outs of basketball and even how to swim.

Peter (45), an investment banker, resides in China with his partner. Their 2‐year‐old twin girls were born in Los Angeles using the surrogacy process. His partner, an American citizen, is engaged in international trade, and together, they chose Shanghai to be their home. In their household, Peter is affectionately referred to as ‘Daddy’ by the twins, while his partner, David, is fondly called ‘Mommy,’ a testament to their distinct personalities. This familial arrangement reflects their unique dynamic and the roles they have embraced. Peter says,

Around here, I am known as ‘Daddy’ or ‘Papa,’ and my partner David is ‘Mommy’ or ‘Mama.’ I am the one who teaches life lessons like playing with them or teach the twins on reading stories, drawing; while David is the one who kisses the boo‐boos and makes them yummy meals. Every day, I hear these little voices calling out, ‘Hey, Daddy!’ and there is nothing quite like it. It is pure joy interacting with my little angels. They are the cutest things you have ever seen. I am head over heels for them. Being a dad, especially a gay dad, is a rollercoaster, you know? Ups, downs, twists, turns, the works. My daughters see me as their dad, and I'm the one who's planning for their future education abroad. On the other hand, David is like their mom, always there to take care of their emotional needs.

These gay fathers exhibit acute awareness of the intricacies of parenthood ‐ the bedtime narratives, the healing of minor injuries, and the whispered dreams. Peter's experience vividly denotes parenthood. It is an intricate novel, a rich tapestry interwoven with love, chaos, and indelible sticky fingerprints. The parents of gay couples often show support, especially the mothers, who embrace the role of grandmother. According to Bourdieu's field theory, individuals navigate social fields by accumulating and deploying various forms of capital. In this context, the desire to have children can be seen as a strategic move to accumulate social and cultural capital. By raising children who are well‐educated and socially adept, upper‐class gay individuals reinforce their own positions within the social hierarchy. This finding supports Bourdieu's assertion that family practices are often influenced by the desire to maintain or enhance social status and raise their children in a more liberal way.

### 5.1 Hiring Nannies (Aunties)

Bourdieu's field theory suggests that individuals use various forms of capital to navigate social fields and maintain or enhance their social positions. In this context, hiring a nanny can be seen as a strategic move to deploy economic and cultural capital in ways that benefit their children's development and social standing. By providing their children with high‐quality care and early educational experiences, upper‐class gay individuals reinforce their own positions within the social hierarchy and ensure the transmission of capital to the next generation.

Yuyang (34), an electrical engineer, and Xiaoyu (28), an architect, reside together in Beijing. They have been in a committed relationship for 9 years. They share the desire to start a family. Yuyang, an alumnus of Harvard University with a PhD, is currently employed at a publicly listed company in Beijing. Yuyang hails from a family with a thriving business. His mother is a cadre member with robust political and economic networks. He initially studied in San Francisco, California, and eventually moved to Boston, where he completed his doctorate in electrical engineering at Harvard. In 2023, following the relaxation of the birth certificate policies, Yuyang embarked on regular trips to Los Angeles to test and prepare for having twins through CS. Yuyang and Xiaoyu welcomed twins Sam and Saffron in December 2023. Their resources, including a nanny, helped them balance career and parenthood effectively. They believed their twins would significantly benefit from having an excellent nanny. Yuyang says,

I went through ten bad aunties before I got a good one. This one has a secret talent: She knows precisly which lullaby will put the baby to sleep fastest. Auntie changes diapers with the grace of a ballet dancer. No leaks, no fuss—just a swift swoop. She even folds those tiny diaper tabs into origami swans. Auntie orchestrates naptime like a symphony conductor. She hums soothing melodies, rocks the crib gently, and whispers tales of magical dreamlands. Bonus points if she can put the baby to sleep while balancing a book on her head. Auntie transforms our home into a baby‐safe wonderland. Electrical outlets? Covered. Sharp corners? Padded. Stairs? Fortified with invisible baby gates. She is the reason our coffee table now has foam corners. Safety first, style second.

Ruiji (37) is an entrepreneur in a listed company in Tianjin, but he is single. He has a 2‐month‐old son and uses a shared approach; one auntie oversees daily chores and responsibilities, and another auntie—his relative—focuses on education. The aunties essentially serve as co‐parents, helping educate and care for the son while he maintains parental oversight, embodying a communal child‐rearing model. Ruiji says the two nannies are incredible and become their household superheroes. He says,

The nannies take care of everything—light housekeeping, whipping up delicious meals, and making sure our little one is safe and comfy. She is like a magical blend of Mary Poppins and Alfred Pennyworth (minus the butler outfit). I asked her if she would stay overnight to take care of late‐night diaper changes and cuddles that happen when the moon is high. I was so glad she said yes! I like to sit with my son for story time or playtime with him. Together, the Aunties and I are like a well‐oiled childcare machine. They are gems…

All the gay fathers interviewed employed at least one nanny to aid in child‐rearing and 10 out of 35 employed two. The term “nanny” (auntie) is used broadly to include any middle‐aged female domestic housekeeper. The “nanny” resides in the employer's home and is typically granted one statutory holiday per week, usually during the weekend or as per the agreement between the employer and them. This arrangement mirrors the domestic maid system prevalent in Hong Kong. Such an arrangement allows gay fathers to form their desired families while delegating caregiving responsibilities to these caretakers, a strategy that becomes particularly crucial given the additional challenges they face.

## 6 Relational Selfhood Intertwined With Parents

Relational selfhood refers to the concept that an individual's identity and actions are shaped by their relationships with others. For upper‐class gay elites in China, their selfhood is deeply intertwined with their parents and broader family networks. This relational selfhood influences their approach to raising surrogate children. For instance, all the gays kept saying their parents' support and involvement are crucial in their child's upbringing. Parents provide not only emotional support but also practical help. This highlights how relational selfhood is embedded within familial contexts, where parents and extended family members play a significant role in child‐rearing. Sam (24, entrepreneur) is the founding director of a listed company in Shanghai. Now his son is 4 months old. Sam's approach to parenting is heavily influenced by his own upbringing and the values instilled by his parents. Sam's mother gave birth to him when she was only 20. Therefore, his mother suggested that she could help Sam by becoming the surrogate mother. Sam says,

My mom (aged 44), bless her heart, came up to me one day and said, “I will be your surrogate mother. It will save you a ton of money.” I was like, “Wait, what?” She told me to find a surrogacy agency, look for egg donors in egg banks, combine them with my sperm, and implant them in her womb. It was a wild idea. But you know what? My mom is not my surrogate mother, but I found a surrogacy agency right here in Shanghai and implanted the embryos into the body of a local surrogate mother! Ten months later, I am holding my baby girl in my arms. My mom was over the moon and could not stop smiling. And now, she is helping raise and educate my little one. Crazy, right? But that is family, and I have a great mom!

Haoyi (40) lives in Tianjin and works with the Chinese government. He had a son who was only 1 month old when I interviewed him in January 2024. Haoyi says,

My dad and mom are like my right hand when it comes to looking after my son. We had a big fight and intergenerational conflicts when I came out to them 20 years ago. However, they have got my back. They are there, teaching and caring for the little prince. You can see the joy in my dad’s eyes. Just the other day, my son had a cold, and mom was there, giving him his medication. Suddenly, she says she is ‘the mom’ and I am ‘the dad.’ Can you believe that? I mean, I work at this investment bank. There is not much time for family, but I do my best. I focus on what is important: My kid’s education and his upbringing. It is a team effort.

For gay fathers, the longing for parenthood transcends mere biological impulses; it becomes a grammar of behaviour—an innate language absorbed without conscious effort. This internal compass, akin to a native language, shapes their yearning for parenthood beyond mere biological imperatives (Yan 2021). Habitus influences gays' narratives of love, longing, and legacy with their class‐based capital. Habitus is not only internalized by gay individuals or couples but also influenced and internalized by their parents' support. Kei (58) is the father of one gay and supports his son. He says,

I am always very liberal to my son after he came out to me. We are committed to supporting him at every turn. We are there for doctor’s appointments, baby showers, and late‐night feedings. We are there to commemorate the milestones and overcome when challenges arise. As a father, I have a wealth of experience and knowledge. I navigate societal expectations, address any concerns our relatives may have, and ensure that our grandchild is raised in a loving and accepting environment, always with open arms.

Ying (55), a mother of a gay individual, was devastated when she found out her son was gay. However, after 5‐years of walking with her gay son, she has a better understanding of his needs. She became more supportive and sympathetic toward her son, who wanted a family by CS. With Ying's well‐educated or have a broad understanding of the world can pass on her capital to support him, to navigate societal norms and expectations, and to provide emotional and financial support to their children. Ying was able to procure a baby girl who was 4 months old at the time of the interview. Ying's mother says,

If I choose not to stand by him, I stand to lose both my son and my granddaughter. But if I decide to support him, I get to keep my son and granddaughter close. We’re in this situation as a team, jointly raising his little girl. I do my part ‐ whipping up meals, keeping the house tidy, caring for the baby. We have these precious moments, you know. It is remarkable how our relatives refrained from inquiring about every detail of the baby. They might have surmised that my son is gay and that the baby girl was born through surrogacy. Yet, they did not utter a single negative word or attempt to hinder us. I suppose when everyone lays eyes on the little darling, they refrain from probing further. The girl is a beacon of luck in our family.

Another father, Wing (59) shared the same experience with Ying. He says,

I was once a man of hot temper. My son and I found ourselves embroiled in a silent feud that lasted for months. It felt as though I had lost him when he departed for the USA… However, I made a conscious decision to change. Embracing the principles of a liberal father, I became more open‐minded and accepting. This transformation in my attitude marked a new chapter to accept his decision, including his surrogate children. I am so happy to enjoy my stay‐at‐home granddad!

Another mother, Man (59), talked about her 2‐month‐old grandson and said he is the bridge in her family. Man says,

The role of a “mother” is crucial to my grandson, and I am delighted to fill this role. My son and I had heartfelt conversations that stretched into the late hours, discussing everything from diaper brands to the ideal theme for the nursery. I have been there for him, mastering feeding schedules and diaper changes and weathering those sleepless nights. I recall teaching him how to cradle his little son and how to burp him after feeding. My siblings and other relatives have been supportive, showering my grandson with gifts, milk powder, and toys. I leveraged my personal influence and network to assure my family that my son doesn’t need to feign a desire to marry a woman. This is his truth, and it should be respected and accepted.

The verbatims above underscore the unique joy and continuity that grandparenthood brings to the parents of their gay sons. These grandchildren serve as a conduit, bridging the generations and fostering a sense of continuity and perpetuity as parents witness their family's growth and progression. In the context of queer communities, these children are bonding and bridging within family and kinship structures within families and kinship networks (Bourdieu 1986). Gay parents' involvement and the class‐based families of the upper‐class families allow them to focus on providing a well‐rounded upbringing for their surrogate children, including access to the best education and cultural experiences. This dynamic interaction underscores the importance of considering both individual agency and structural factors in understanding parenting practices and child‐rearing strategies.

## 7 Field‐Intertwined With the State Policy

Governmental policies and interventions also play a significant role in shaping parenting practices and child‐rearing strategies. Policies related to education, childcare, and family support can either facilitate or constrain gays' ability to accumulate and deploy capital. The relaxation of children's certification requirements since 2023 has made it easier for gays to access certain services and opportunities for their children without the previous bureaucratic hurdles. This demonstrates how state interventions can impact the field in which parents operate, influencing their strategies and decisions. Kuanxi (39) lives in Beijing. He works for a listed film company in Beijing. Kuanxi said that he really loves kids and longed to be a parent. In 2015, he found an underground surrogacy centre in Beijing and had a girl. In 2024, the girl, now 8, was attending primary school in Beijing. He had difficulty obtaining a *hukou* in 2015 and eventually had to bribe the cadre working in the birth certificate bureau. However, with the recent policy change, Kuanxi said getting his second child was much easier.

Eight years ago, my daughter was this tiny bundle of hope, a gift from an underground surrogacy centre. Birth certificates were like rare treasures, locked away in bureaucratic vaults. I had to bribe a cadre to secure my first child. It felt like a secret pact with destiny. However, in 2023, the winds shifted. The Great Wall of bureaucracy is crumbling. With my second child, it is much easier. Birth certificates? They are no longer locked away; they are like confetti at a New Year’s celebration.

Qiang (38) reminisced that it was a nightmare for him to get the birth certificate and *hukou* for his 10‐year‐old son in 2013. Qiang says,

The Party said, “Let love bloom.” So, they relaxed the rules. Gay father is no longer in the shadows. We are sunflowers turning toward the light. We hold our children’s hands and walk through *hukou* doors. Our stories ripple across Beijing’s lakes and whisper through the *hutong* (alley). And guess what? The People’s Daily poll? My gay friends all say, “Surrogacy? Why not? We are rewriting the script. One birth certificate at a time. Now, surrogacy is not a secret handshake; it is a high‐five.”

The societal norms, or the “rules of the game,” have been redefined, with the LGBTQ + communities overcoming obstacles to parenthood. China, upon introspection, recognized its own image—a nation in dire need of increasing its workforce. Despite the illegality of underground surrogacy in China, the government tacitly acknowledges the need to change. Therefore, they look the other way, allowing the practice to persist. The government's stance is pragmatic: regardless of the means, if the result is beneficial—if the “cat catches the mice”—then the method is acceptable. China has come to understand that the concept of family is multifaceted, encompassing traditional, unconventional, and diverse forms. CS, once considered taboo, is now viewed as a way toward the future. This shift reflects China's evolving societal attitudes and its commitment to addressing its demographic challenges.

## 8 Discussion and Conclusion

I use field theory to illustrate how gay fathers facilitate their class‐based capital and their dialectal relationship with their family of origin to have their surrogate children. Bourdieu's field theory suggests that upper‐class gay individuals navigate social fields by accumulating and deploying various forms of capital. In the context of raising children, relational selfhood, parental influences, and governmental policies collectively shape the strategies parents use to enhance their children's social and cultural capital. The self and their parents collaborate to form a harmonized and optimized family (X. Qi 2016; Tsang 2020b). Upper‐class gay individuals who form families in China through CS can be analysed from three perspectives.

First, the class‐based cultural capital and habitus highlight their proactive approach to pursuing personal autonomy and interests, navigating the neoliberal rhetoric of individual responsibility (Yan 2021; Tsang 2019) was nonexistent in the 1970s. However, Today's China has not fully embraced neoliberal thought, opting instead for a selective adoption of market‐based principles. This has created a unique socio‐economic environment where gay individuals from the single‐child generation exhibit more individualistic life orientations. Yet, they continue to uphold nuanced intergenerational obligations and public manners rooted in traditional values. This duality reflects a complex interplay between modern economic influences and enduring cultural norms, necessitating a nuanced understanding of China's socio‐economic landscape in contemporary academic discourse. The gay individuals leverage their class‐based capital, creating space to form families with support from their parents and nannies. They entangled within a wider web of relationships to nurture and socialize their surrogate children with their family of origin (Lin et al. 2017; Tsang 2020c). Aside from certain cadres who must maintain discretion regarding their sexual orientation, gays of the LGBTQ + community can freely express their identities. Their parents and nannies often undertake the motherhood role, alleviating the tension within the ontological structures of motherhood. In the context of nurturing and educating surrogate children in China, gays leverage their class‐based cultural capital and habitus to provide their children with a broad range of educational opportunities, socialization, and parenting. They assign parental roles based on their character and gendered specialties and nurture their children via diverse cultural experiences, international perspectives, and advanced educational resources. This fosters a cosmopolitan habitus in their children from an early age. Gay and their parents with substantial cultural capital are likely to value education and invest significant resources in their children's learning. This can lead to a cycle where cultural capital and the associated habitus are passed along from generation to generation, enabling them to fulfil China's heteronormative family‐centred culture. In the case of surrogate children within gay families, research indicates that these children function as well as, if not better than, children in the general population (Green et al. 2019). Surrogate children raised by gay fathers via CS had significantly fewer externalizing problems (such as aggression and rule‐breaking) and internalizing problems (such as anxiety and depression) than a comparison sample of children drawn from a normative population (Green et al. 2019).

Second, this research suggests that China's liberalization of its birth rate and birth certificate policies herald a new era for upper‐class queer communities. Their surrogate children are conduits, communicating societal expectations of marriage and the formation of heteronormative families. The institutional expediency of having children is intertwined with the broader social environment (Zhang and Ong 2008). Gays have capitalized on these policy shifts to embrace parenthood and openly disclose their sexual orientation. This study advocates for a broader implication, suggesting that parents should adopt a liberal stance in accepting their gay sons when they disclose their sexual identity. The education of parents to accept their queer children is a crucial measure in mitigating further harm to *tongqi* who unwittingly marry a gay husband (Tsang 2021a, 2021c), who may be involved in fraudulent marriages, or lesbian women engaged in marriages of convenience.

Third, by relaxing *hukou* and birth certificate regulations, the Chinese government has implemented specific measures that help the queer community. These policy modifications were made to address the country's declining birth rate and a demographic crisis requiring a rethinking of family planning policies. This approach could potentially address the issue of ‘black‐market children’ – denote children born in contravention of the China's one‐child policy (Zeng and Hesketh 2016). Those children may lack birth certificates or identification documents, resulting in the denial of fundamental rights such as education, healthcare, and formal employment, or even utilise high‐speed train services. However, it is essential to note that these policy benefits may be limited to the upper‐class queer communities, who can use their class‐based capital, *habitus*, and field (policy) to achieve these desired life outcomes. This raises questions about the inclusivity and equity of these policies and highlights the need for the government to ensure that all members of the queer community may benefit from these changes regardless of status.

In conclusion, the parenting practices of gay fathers in China are significantly influenced by their class‐based capital, which encompasses economic, cultural, and social resources. These fathers leverage their economic capital to provide high‐quality care and educational opportunities for their children, ensuring a stable and nurturing environment. The cultural capital they possess, often reflected in their educational backgrounds and professional achievements, enables them to navigate societal expectations and advocate for their children's well‐being. The social capital inherent in their kin networks plays a crucial role in their parenting strategies. Bonding within the family unit is strengthened through the active involvement of grandparents and extended family members, who offer emotional support and practical assistance. This intergenerational support system not only reinforces familial ties but also provides a robust foundation for the children's development. Overall, the interplay of economic, cultural, and social capital enables gay fathers in China to effectively raise their children, despite societal challenges. By fostering strong family bonds, leveraging kin networks, and utilizing their class‐based resources, these fathers create a supportive and enriching environment for their children's growth and development. Future policies and societal changes should continue to recognize and support the diverse family structures within the LGBTQ + community, ensuring that all children have the opportunity to thrive.

## Data Availability

Research data are not shared.
